# Higher genome mutation rates of Beijing lineage of *Mycobacterium tuberculosis* during human infection

**DOI:** 10.1038/s41598-020-75028-2

**Published:** 2020-10-22

**Authors:** Mariko Hakamata, Hayato Takihara, Tomotada Iwamoto, Aki Tamaru, Atsushi Hashimoto, Takahiro Tanaka, Shaban A. Kaboso, Gebremichal Gebretsadik, Aleksandr Ilinov, Akira Yokoyama, Yuriko Ozeki, Akihito Nishiyama, Yoshitaka Tateishi, Hiroshi Moro, Toshiaki Kikuchi, Shujiro Okuda, Sohkichi Matsumoto

**Affiliations:** 1 Department of Bacteriology, Niigata University School of Medicine, 1-757, Asahimachi-Dori, Chuo-ku, Niigata, Niigata 951-9510 Japan; 2 Department of Respiratory Medicine and Infectious Disease, Niigata Graduate School of Medical and Dental Sciences, 1-757, Asahimachi-Dori, Chuo-ku, Niigata, Niigata 951-9510 Japan; 3 Department of Bioinformatics, Niigata University School of Medicine, 1-757, Asahimachi-Dori, Chuo-ku, Niigata, Niigata 951-9510 Japan; 4 Department of Infectious Disease, Kobe Institute of Health, 4-6-5, Minatojima-nakamichi, Chuo-ku, Kobe, 650-0046 Japan; 5 Department of Infectious Disease, Osaka Prefectural Institute of Public Health, 1-3-69, Nakamichi, Higashinari-ku, Osaka-shi, Osaka 537-0025 Japan; 6 Clinical and Translation Research Center, Niigata University Medical and Dental Hospital, 1-757, Asahimachi-Dori, Chuo-ku, Niigata, Niigata 951-9510 Japan; 7 Department of Respiratory Medicine, Graduated School of Medicine, The University of Tokyo, 7-3-1, Hongou, Bunkyo-ku, Tokyo, 113-8655 Japan; 8 Laboratory of Tuberculosis, Institute of Tropical Disease, Universitas Airlangga, Kampus C Jl. Mulyorejo, Surabaya, Indonesia

**Keywords:** Bacteria, Microbial genetics

## Abstract

*Mycobacterium tuberculosis* (*Mtb*) strains of Beijing lineage have caused great concern because of their rapid emergence of drug resistance and worldwide spread. DNA mutation rates that reflect evolutional adaptation to host responses and the appearance of drug resistance have not been elucidated in human-infected Beijing strains. We tracked and obtained an original *Mtb* isolate of Beijing lineage from the 1999 tuberculosis outbreak in Japan, as well as five other isolates that spread in humans, and two isolates from the patient caused recurrence. Three isolates were from patients who developed TB within one year after infection (rapid-progressor, RP), and the other three isolates were from those who developed TB more than one year after infection (slow-progressor, SP). We sequenced genomes of these isolates and analyzed the propensity and rate of genomic mutations. Generation time versus mutation rate curves were significantly higher for RP. The ratio of oxidative versus non-oxidation damages induced mutations was higher in SP than RP, suggesting that persistent *Mtb* are exposed to oxidative stress in the latent state. Our data thus demonstrates that higher mutation rates of *Mtb* Beijing strains during human infection is likely to account for the higher adaptability and an emergence ratio of drug resistance.

## Introduction

Tuberculosis (TB) is one of the top 10 causes of death and the leading cause from a single infectious agent above HIV/AIDS^1^. TB generally has two clinical states comprised of frequent long-term latency and active disease. Approximately one quarter of the world’s human population is asymptomatically infected with *Mycobacterium tuberculosis* (*Mtb*), a state called latent tuberculosis infection (LTBI)^1^. LTBI is a large source of the disease and generally, 5–10% of LTBI population develops active TB during lifetime^2^.

*Mtb* spread out of Africa over 70,000 years ago, following the early human exodus from Africa^3^. The current global *Mtb* spread can be classified into seven lineages^4^. Lineage 4 and 2 which spread mainly to Europe and Eurasia respectively, are known to be highly pathogenic^4,5^. In particular, *Mtb* Beijing lineage belonging to lineage 2 has received much attention due to its strong tendency to become highly pathogenic, drug resistant, and highly transmittable^5–10^. Recent studies reported that many genes, including drug target genes tend to be mutated in Beijing strains^11^, and are associated with multi-drug resistance (MDR) outbreaks^12–14^.

It is known that the genomic mutation rate of *Mtb* is 10–100 folds lower compared to other pathogens^3,15^. Since the insertion of foreign genes into the *Mtb* genome is rare, it is thought that evolutional acquirements of pathogenicity and drug resistance mainly depend on mutations in the genome. Genomic mutations result from errors in DNA synthesis, and are therefore likely to accumulate during *Mtb* division in active TB. On the other hand, in non-dividing *Mtb* during latent state, genomic DNA can be damaged by the host responses and be mutated by erroneously repairing.

It is hard to experimentally verify rates of *Mtb* replication and its genomic DNA mutation during latency. However, Ford et al. reported a curve, describing mutation rates as a function of replication, which calculates mutation rates by measuring the number of chromosomal mutations during a defined period of infection^16^. Using *Mtb* infection model in rhesus macaque, they showed that the generation-time versus mutation rate curves of *Mtb* were similar regardless of the length of the latent period^16^. On the other hand, Colangeli et al^17^ reported higher mutation rates of *Mtb* isolates that developed TB within two years compared to *Mtb* isolates that progressed to disease more than two years post-infection. In both cases, *Mtb* strains of lineage 4 were used. However, the genomic mutation rates of *Mtb* strains of Beijing lineage during human infection has not been elucidated so far.

**Figure 1:**
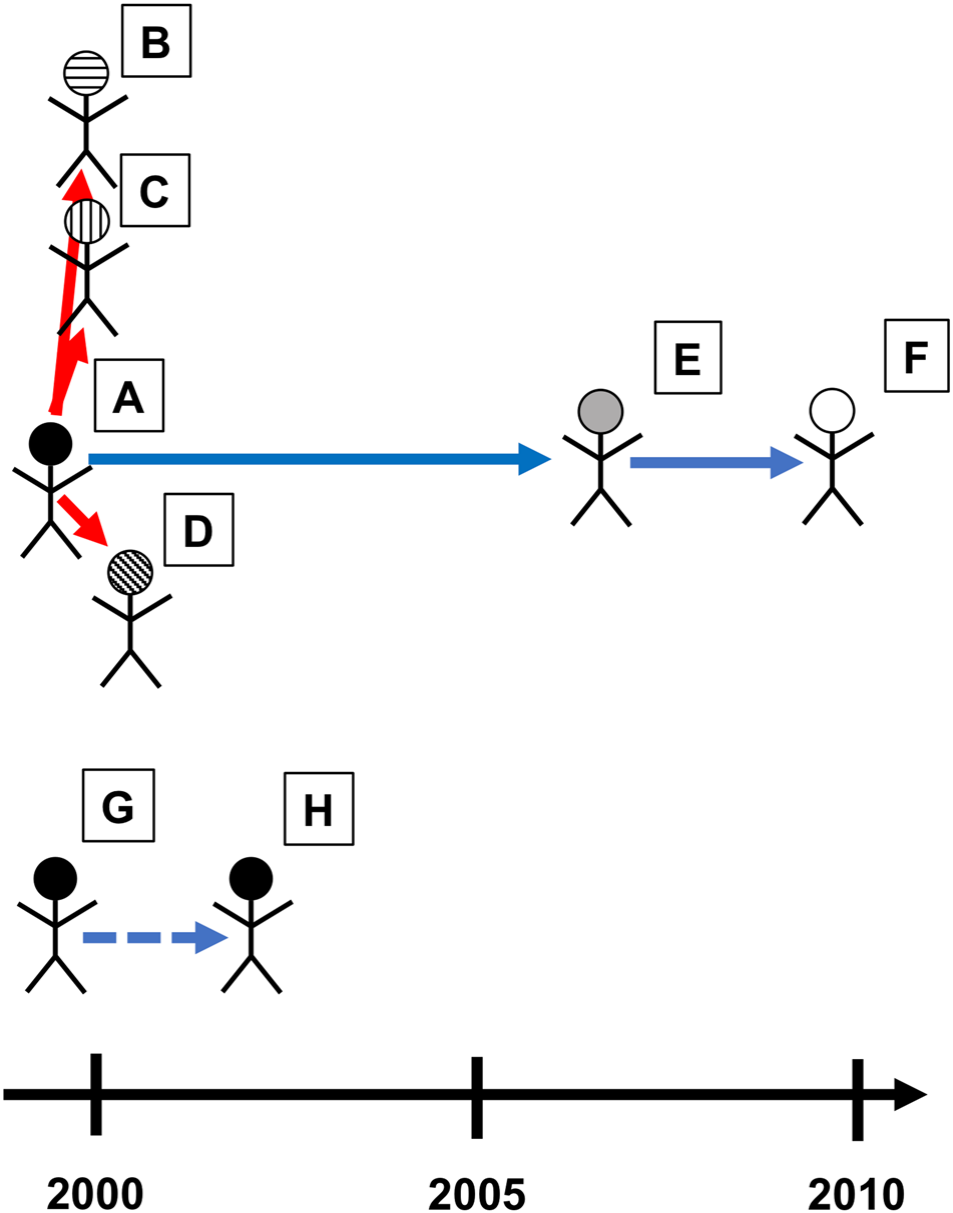
Epidemiological relationships among eight TB cases. Chronological representation of each subject of the TB outbreak analyzed in this study. Each square represents a subject who developed active TB. Red and blue lines represent the direction of transmissions that led to TB development within one year after infection and more than one year after infection, respectively. The blue diagonal line means that a subject relapsed TB more than one year after he was cured.

We obtained one original strain that caused the *Mtb* outbreak in Japan and five other isolates transmitted by patients and two isolates from the patient before and after causing recurrence (Fig. 1). Three out of the five transmitted isolates were derived from patients (rapid-progressor, RP) who developed disease within one year after infection, and the other three isolates were obtained from patients (slow-progressor, SP) who developed TB more than one year after infection.

In this study, we performed whole genome sequencing of these eight isolates in order to know the propensity and rate of genomic mutations of *Mtb* Beijing lineage during human infection.

## Results

### Epidemiological relationships among eight *Mtb* Beijing isolates

We selected six *Mtb* Beijing isolates from the strain bank in Kobe Institute of Health. These isolates cover the events of latency and active transmission through sequential hosts (Fig. 1). Five of them were obtained from patients who were involved in the TB outbreak at a junior high school in 1999. Isolate A was from the initial index-case patient of the outbreak, Isolate B and Isolate C were obtained from patients whose TB onset was 2 months after the initial index case. Isolate D was obtained from a patient who received LTBI treatment of INH but developed active INH-resistant TB during therapy. Isolate E was selected as the latency transmission case of the outbreak. The patient (Subject E) was a classmate with the initial index-case patient (Subject A), likely exposed in the 1999 event, and developed active TB 7 years 6 months later after the index case. Subject E caused another outbreak in 2006 at a hair salon where the patient worked. The patient (Subject F) was a customer at the hair salon, likely exposed in the 2006 event, and developed active TB 2 years 9 months later in 2009. All isolates from the six patients demonstrated an identical variable numbers of tandem repeats (VNTR) genotype.

We selected the other two *Mtb* Beijing isolates from the strain bank in Osaka Institute of Public Health. This patient (Subject G) was diagnosed as pulmonary TB with rifampicin resistant *Mtb* isolate (Isolate G) for the first time in 1999. After six months treatment and getting cured, he had a relapse in 2001 with *Mtb* isolate (Isolate H) resistant to INH, rifampicin, streptomycin and ethambutol. Even though these isolates showed different drug sensitivity patterns, they had identical VNTR genotype and belonged to the Beijing lineage.

Mutation rates were compared between the two groups; early-onset patients who developed TB within one year (rapid-progressor, RP), such as, pairs A-B, A-C and A-D, and those who developed or relapsed after a latent period of more than one year (slow-progressor, SP), such as, pairs A-E, E-F and G-H.

### Determination of genomic mutations

First, low quality reads were removed with the Usearch parameter maxee 0.5 recommended for quality control of FASTQ files. Subsequently, the reads sequences were mapped to the *Mtb* H37Rv genome sequence as a reference with BWA, because it is refined genome data the closest of the published genomes to the strains we analyzed. GATK was used for mutation calling and about 1600 mutations were found in each strain (Filter 1 in Table 1). Second, consensus genome sequence of Isolate A, which was derived from the initial index case, was constructed based on only high quality SNPs filtered with the VCF parameters described in “Methods” section, because of the following processes. The reads sequence in each strain was re-mapped to the consensus genome sequence and Indels and SNPs were re-called (Filter 2). Next, indels re-called in Isolate A were removed in each strain, because they were regarded as the same as the Indels originally called when using H37Rv as the reference (Filter 3). Higher quality mutations were extracted based on the VCF parameter filtering (Filter 4). Finally, mutations found in repeat sequence of the PE-PGRS family were filtered (Filter 5). As a result, 118 mutations in Isolate B-F were detected (Supplementary Table S1). There were total of six single nucleotide polymorphisms (SNPs) with amino acid substitutions (Supplementary Table S2).

**Table 1:** The numbers of single nucleotide polymorphisms differences among each isolate.

Isolate filter*	A	B	C	D	E	F
1	1645	1616	1607	1603	1632	1628
2	329	439	416	413	442	425
3	185	296	274	272	299	282
4	8	146	160	56	53	56
5	0	107	108	47	48	46

*1: maxee 0.5, reference is *Mtb* H37Rv.

2: Consensus sequence of Isolates A limited SNP.

3: Excepted insertion and deletion of Isolate A.

4: Filtering by vcf parameters.

5: Excepted repeat sequence, and consensus sequence of Isolate A.

Isolate B and C possessed more SNPs in upstream regions of genes and synonymous mutations than the other isolates (Table 2). The number of the missense variants that can change amino acids were similar in each strain despite of the duration of incubation period (Table 2).

**Table 2:** Patterns of single nucleotide polymorphisms differences between each isolate.

Isolate	A	B	C	D	E	F
Total	0	107	108	47	48	46
Upstream gene variant	0	15	16	8	5	6
Missense variant	0	2	2	2	4	3
Disruptive inframe deletion/insertion	0	0	0	0	0	0
Synonymous variant	0	89	90	36	38	37
Frameshift variant	0	1	0	1	1	0
Stop gained	0	0	0	0	0	0
Conservative inframe deletion/insertion	0	0	0	0	0	0
Splice region variant and stop retained variant	0	0	0	0	0	0
Stop lost and splice region variant	0	0	0	0	0	0

SNPs and indels in drug resistance genes were only found in Isolate D, which had a large-deletion including *katG* (Supplementary Fig. S1). *katG* encodes catalase peroxidase, an enzyme that converts isoniazid (INH) to its biologically active form^18^. INH resistance commonly occurs due to mutations in the katG gene^19^. In this case, this large deletion in the katG gene region accounts for INH resistance in Isolate D. Large deletions with more than 1 kb were explored in all strains. As a result, we detected 18 large deletions. Of these, 17 large deletion sites were common in Isolate A to Isolate F. Thus, these large deletions were regarded as the regions that existed in the ancestral strain (Isolate A).

### Analysis of mutation positions

We analyzed whether there is a tendency in the location of the mutation on the genome in five strains of the outbreak case (Supplementary Fig. S2). When comparing the number of mutations in every 200,000 bp (or 100,000 bp), whether the number of mutations was the same as the average number of mutations throughout the genome was tested by Mann–Whitney U-test. Since the *p* value did not fall below 0.05 (*p* > 0.2), and is not different from the average of the whole genome, the sites of mutation were considered to be unbiased. There was also no tendency in the mutation location on the genome between RP and SP groups. Next, we assessed mutations related to the length of the latent period. In the SP group, the same mutant genes found in isolates E and F were Rv0800 and Rv1165. Rv0800 which is involved in intermediary metabolism and respiration had an amino acid substitution. However, it is likely that the mutant gene was transmitted from subject E to F, because the same mutations in Rv0800 were observed in Isolate E and F. We also could not find intergenic regions significantly associated with latent periods, consistent with previous reports^16,17,20^.

### Phylogenetic analysis

**Figure 2:**
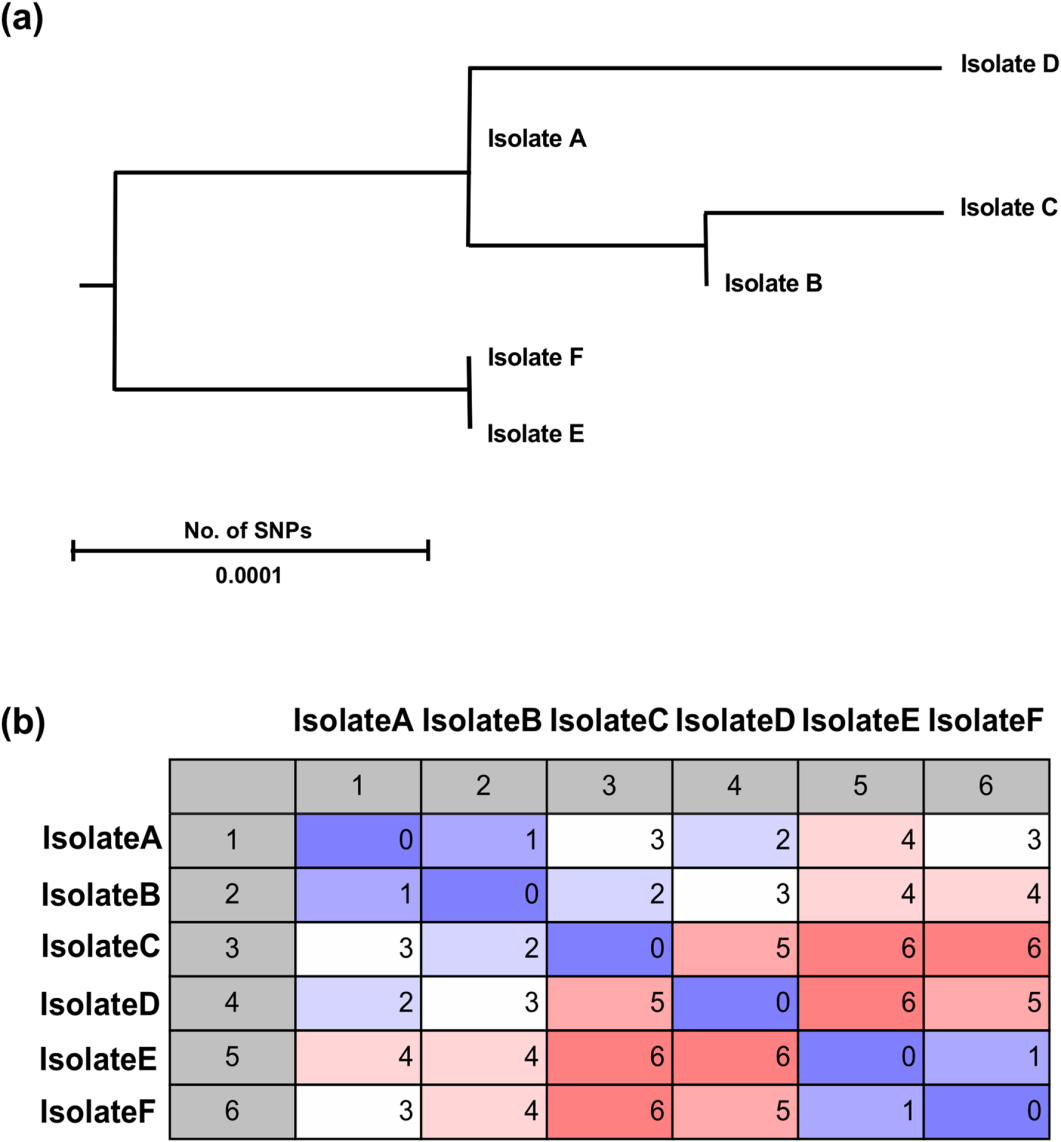
Phylogenetic analysis of *Mtb* Beijing strains isolated from a cluster of cases in Japan. Phylogenetic analysis was performed using CLC Genomics Workbench (QIAGEN Aarthus A/S). (**a**) Evolutionary tree rooted on *Mtb* H37Rv using SNP-based phylogenetic analysis. (**b**) SNP matrix. This represents the evolutionary distances among strains.

A phylogenetic analysis of all SNPs from the six *Mtb* Beijing strains from the outbreak case was performed. The analysis supported the epidemiological connections identified between isolate A-B, A-C, A-D, A-E, and E-F. This analysis further confirmed that the ancestry of isolate B, C and D were closer than that of isolates E and F (Fig. 2a). Indeed, SNP distances of isolate C-E, B-E, and B-F were longest and which of isolates A-B and E-F were shortest (Fig. 2b). These results support our hypothesis that the trend of the two groups (RP and SP group) is different genetically.

### Mutation rates among *Mtb* isolates in the outbreak case

In vitro*,* generation time of *Mtb* is approximately 20 h under nutrient-rich conditions^21^. In order to compare the mutation rate of bacteria from each clinical condition, Ford et al. derived a lower limit for the bacterial mutation rate in vivo, which they defined as the predicted mutation rate per generation if the in vivo generation time was equivalent to the in vitro generation time of 20 h, calledμ_20_. They calculated the mutation rate (μ) as described in “Methods” section.

**Figure 3:**
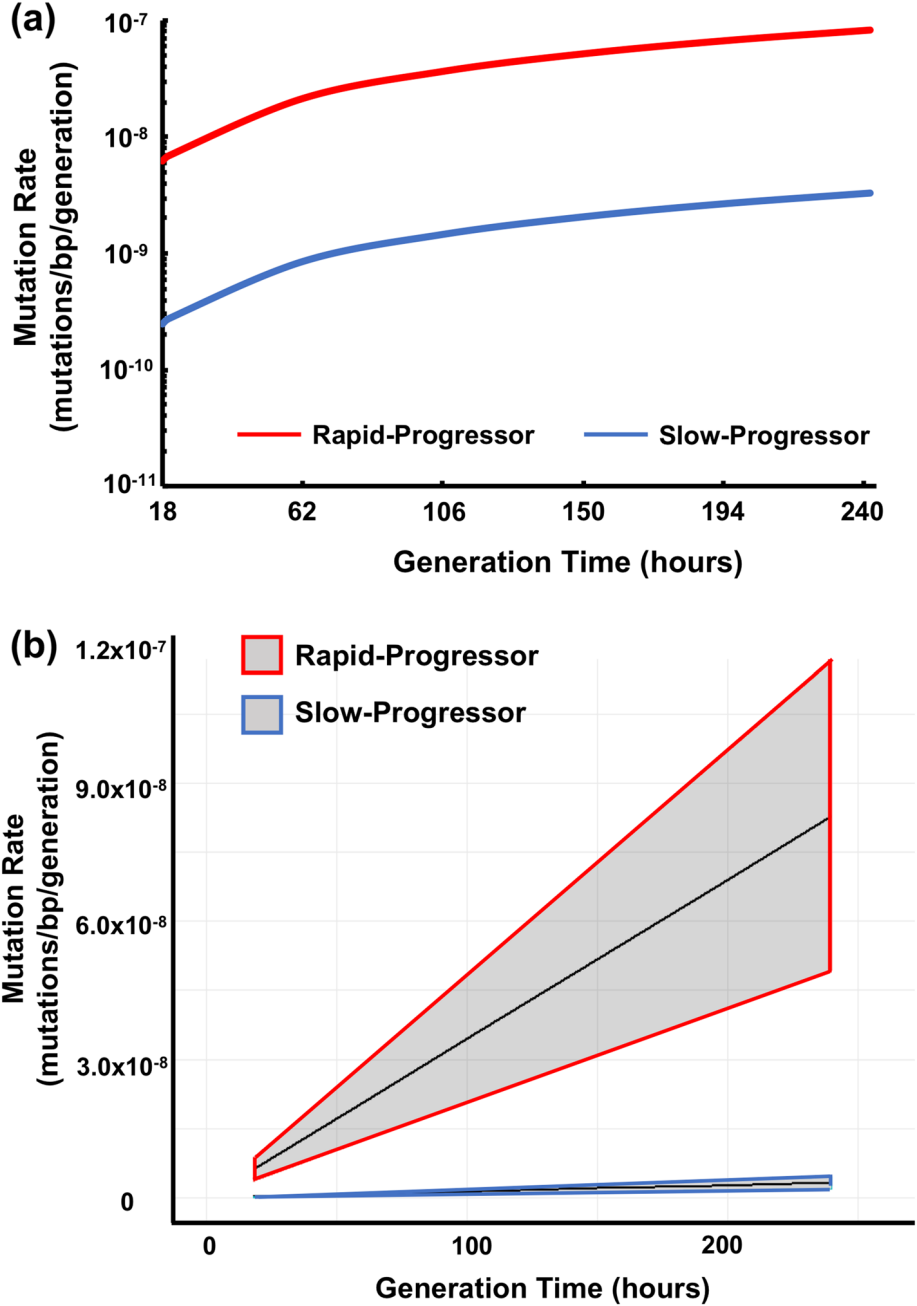
In vivo mutation rates in *Mtb* Beijing strains for generation time ranging from 18 to 240 h. The mutation rate was estimated based on the number of unique SNPs observed in each condition (three isolates from RP group and two isolates from SP group). This calculation was estimated using the Eq. (16) by Ford et al. and performed over a range of generation times. (**a**) The mutation rates for two groups in this study. The Red and Blue line shows the average mutation rate for the RP and SP group, respectively. (**b**) The mutation rates same as panel (**a**) and the grey areas represent 95% confidence intervals. Graphics were generated using the software program (R statistical computing environment, version 3.5.1).

We estimated the mutation rate (mutation/bp/generation) across a broad range of generation times (Fig. 3a), and also derived a mutation rate (μ_20_) at an assumed 20 h generation time (Table 3). We determined the probability of observing μ when g is fixed at any given time to build the probability distribution function around each estimate and to define 95% confidence intervals (Fig. 3b).

**Table 3:** Mutation rates and rate of genetic change of each isolate.

	Isolate	Mutation rate (mutation/bp/generation)	Rate of genetic change (SNPs/genome/year)
Rapid-progressor	B	1.0 × 10^–8^	18
	C	6.9 × 10^–9^	12
	D	3.5 × 10^–9^	6
Slow-progressor	E	3.8 × 10^–10^	0.6
	F	1.7 × 10^–10^	1
	H	1.9 × 10^–9^	3

In tested strains of the outbreak case, the estimated mutation rate (20 h) of RP group showed 3.5–10.3 × 10^–9^ mutations/bp/generation, while the SP group showed lower mutation rates of 1.7–3.8 × 10^–10^ mutations/bp/generation. If a common generation time is assumed, the estimated mean mutation rates per generation of RP group was approximately tenfold higher than those from SP group (Fig. 3a). The areas of 95% confidence intervals from the two groups does not overlap, suggesting that the mutation rate of RP group tends to be higher (Fig. 3b).

**Figure 4:**
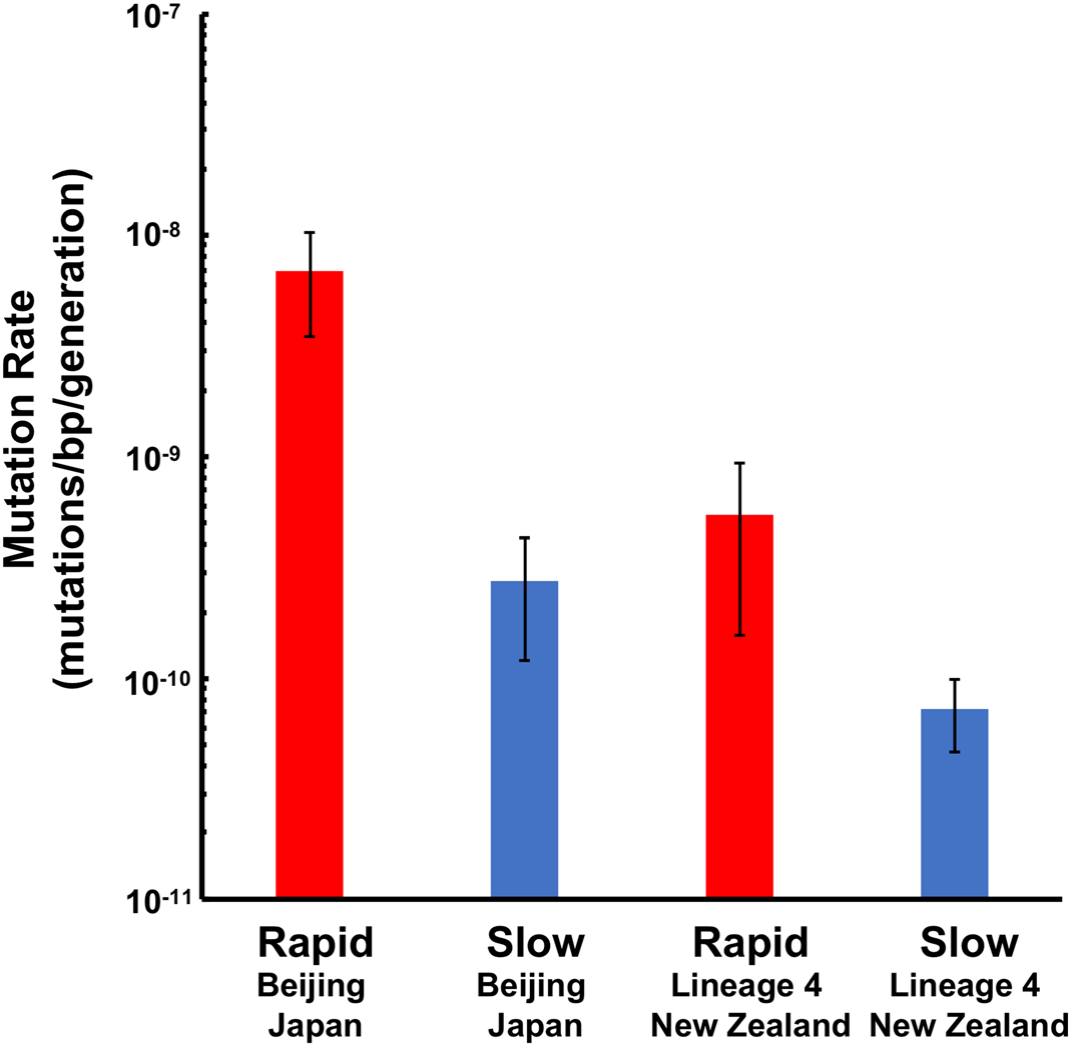
Mutation rate differences between *Mtb* strains of lineage 4 in New Zealand outbreak and the Beijing lineage in Japan outbreak. The mutation rate of each group was calculated using the same method as the Eq. (16) by Ford et al. We compared our data to the mutation rate of lineage 4 in New Zealand, which was reported by Colangeli et al^17^*.*

Secondly, we compared the mean mutation rate (20 h) between *Mtb* Beijing strains of the Japan outbreak case and *Mtb* lineage 4 strains of the New Zealand outbreak case (Fig. 4). The mean mutation rate per generation of *Mtb* isolates from SP group was calculated to be 2.8 × 10^–10^. This was close to the general mutation rate, 2.1 × 10^–10^, which was calculated from *Mtb* Erdman strains infected rhesus macaques^15^. In contrast, the mutation rate of *Mtb* isolates from RP group was calculated to be 6.9 × 10^–9^, showing ten times higher than the general mutation rate. Both mutation rates of these *Mtb* isolates from RP group and SP group were higher than those of *Mtb* lineage 4 strains isolated from the TB outbreak in New Zealand^16^.

In transmission studies^5,16,22–24^, there is an agreement among estimates of 0.3–0.5 SNP changes per year per genome, which are consistent with the estimated 0.2 SNPs per genome per year based on DNA from ancient isolates^25^. The same mutation rate of 0.3–0.5 mutations per year was reported in strains from a primate model with active, latent or reactivation tuberculosis^16^. On the other hand, the rates of genetic change in our study were 6–18 mutations per year in RP group and 0.6–1 mutations per year in SP group (Table 3). These high rates of genetic change suggest that *Mtb* Beijing strains can accumulate an unexpected level of SNP variation in a general situation.

### Higher genome mutation rate of Beijing lineage *Mtb*

Our analysis of the Beijing lineage *Mtb* isolates that caused the outbreak showed an unexpected higher mutation rate. We next assessed whether this higher mutation rate is strain-specific or not.

The data showed that the mutation rate per generation of isolates from SP group in the recurrence case (Isolate H) was calculated to be 1.9 × 10^–9^ and rates of genetic change was 3 mutations per year (Table 3), showing higher mutation rate to that of SP group from the outbreak case. Finally, we compared the mutation rates of all analyzed *Mtb* Beijing strains in this study, 3 of which were RP and the other 3 were SP. Student’s t-test calculation produced a *p* value of 0.029 (95%CI: 9.55 × 10^–10^–1.10 × 10^–8^), which indicates that the mutation rates are significantly different between tested RP and SP groups.

### Polymorphism pattern

**Figure 5:**
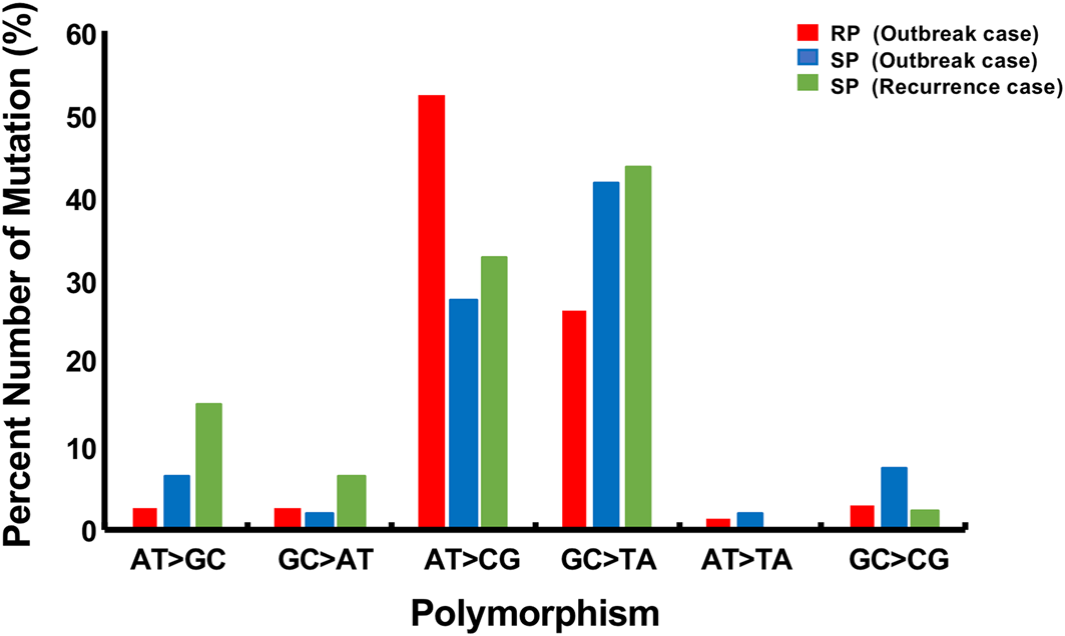
Different type of mutations between RP and SP group. Isolates from RP group (Red) and SP group (Blue) in the TB outbreak case and SP group (Green) in the TB recurrence case were analyzed for different mutation types. GC > AT and GC > TA mutations represent potential products of oxidative damage.

The DNA mutations can be classified as oxidative and non-oxidative damage. Oxidative mutation type was determined by the products of either cytosine deamination (GC to AT changes), or the formation of 8-oxoguanine (GC to TA changes). We counted polymorphism patterns of DNA substitution by the same method defined before^15,16^ and compared the percentage of same mutation patterns in the total mutations of each isolate. *Mtb* isolates from RP group (Isolate B, C and D) possessed more non-oxidative mutation type as AT > GC polymorphism. On the other hand, SP group in the outbreak case (Isolate E and F) and the recurrence case (Isolate H) had more oxidative mutation type seen as GC > TA and GC > AT polymorphisms (Supplementary Table S3 and Fig. 5). We compared the percentage of oxidative mutation type GC > TA between RP and SP including the recurrence case. Student’s t-test calculation produced a *p* value of 0.026 (95%CI: 28.8, − 3.13), suggesting that *Mtb* is exposed to oxidative stress in long term latent infection.

### Analysis of common mutated genes shared by the three studies

We tried to identify mutations related to the length of the latent period shared by three studies (Supplementary Fig. S3). From the New Zealand study^17^, mutation on only one gene Rv2839c was common with our study. However, the mutation did not contribute to the length of the latent period. The Macaques study^16^ on the other hand had one gene, Rv2092c, mutation from an active group similar to our RP group. However, we could not find the genes and inter-genic regions significantly associated with latent periods among the *Mtb* strains of lineage 2 and lineage 4.

## Discussion

Here we demonstrate substantially higher genomic mutation rates of human-infected *Mtb* Beijing strains during both active and latent TB states. We first found that the mutation rates were approximately tenfold higher in early-onset patients than those who developed or relapsed after a long-term latent period of the Beijing strains (Fig. 3). This result was consistent with the previous report that analyzed the human-infected *Mtb* lineage 4 strain^17^ but inconsistent with the study using the rhesus macaque infection model^16^. This discrepancy can be explained by differences in host responses between human and rhesus macaque.

Further, in humans, we found that the mutation rates of Beijing isolates from both active and latent disease phases were higher than each of the mutation rates of lineage 4 strains previously reported^16^. Although both linage 4 and Beijing lineage are highly pathogenic among *Mtb* lineages, our data suggests that the Beijing lineage has potential to rapidly acquire resistance in vivo than lineage 4. Generally, it is thought that the mutation rate of *Mtb* is driven by time dependent rather than replication-dependent manner^26^. Mutations in DNA replication and repair genes are enriched in some *Mtb* strains of lineage 2^27^, but no single point mutation that accelerates the basic mutation rate of *Mtb* in isogenic strains has been reported. *Mtb* Beijing strains of lineage 2 grow avoiding oxidative damage from host immunity^28^, and it may cause more cell division in vivo than *Mtb* strains of lineage 4. Therefore, higher mutation rates in the measured Beijing strains are more likely due to replication errors in active disease state. In contrast, oxidative mutations are frequent in latent state. This suggests the presence of an unknown error prone repairing system in *Mtb* Beijing strains.

Second, we found that the percentage of oxidative damage-related mutations, such as the formation of 8-oxoguanine (GC > TA) and cytosine deamination (GC > AT), were high in SP group (Fig. 5). Similar results were obtained in the study of rhesus macaque infection model^16^. In addition, this is similar to the pattern of polymorphisms that emerged during the evolution of the extensively drug-resistant *Mtb* strains from South Africa^29^. Thus, the mutation capacity of *Mtb* during latent infection as well as the spectrum of those drug resistant mutations suggests that the dominant source of mutation during latency is oxidative DNA damage rather than replicative error^16,30^.

Our WGS data shows evidence that persistent *Mtb* was under oxidative stress during latency. This is consistent with the model that *Mtb* faces to an oxidative environment in the macrophage phagolysosome^18,31^. It is known that the dominant source of mutation during latency is oxidative DNA damage rather than replicative error^30^. This is caused by immune responses during latent infection^32,33^, and by entering into metabolically quiescent state in which DNA replication-dependent error is diminished^34,35^. This suggests that genomic mutation-dependent drug resistance may occur during the latent state. Actually, one isolate acquired isoniazid resistance by the deletion of *katG* during INH-monotherapy (Supplementary Fig. S1).

Resistance to INH is a complex process and has been associated with multiple genes^36–38^. Mutations in codon 315 of *katG* and position-15 in *inhA* promoter region are the most common mutations associated with INH resistance^38,39^. Since *katG* is essential to transform INH into its bioactive form, *katG* deletion causes high resistance to INH^40^. However, *katG* is also important factor for *Mtb* survival depending on its catalase-peroxidase activities^18^. Recent TnSeq data actually indicated that tested all three clinical strains (East Asian lineage, Euro American lineage, Indo-Oceanic lineage) showed requirement of *katG*^41^. This may be the reason why *katG* deletions in INH-resistant strains are less than those with common mutations.

By contrast, *katG* is not essential in *Mtb* H37Rv and higher proportions of *katG* deletion have been reported on occasion^42^. The reason why rare *katG* deletion occurred in Isolate D is likely due to non-essentiality of *katG* in the strain^43–45^. It was reported that loss of *katG* activity was compensated with alkyl hydroperoxide reductase, *AhpC*^37^. Interestingly, it was shown that members of the lineage 2 strain produce a phenolic glycolipid that inhibits the innate immune response^46^, which reduces oxidative burst generated by the host immunity. Such antioxidant property of the strain afforded *katG*-null mutant that exhibits high resistance to INH, especially under strong pressure from INH. Considering these results, our data indicates that part of the increased risk of INH resistance after isoniazid preventive therapy may have resulted from the selection of mono resistant mutants that arise during latency. Especially in Beijing strains of *Mtb*, which has a higher genome mutation rate, isoniazid preventive therapy might cause an increase in drug resistant strains.

Emergence of the *Mtb* drug resistance is mostly dependent on mutations in the genome. And in vitro mutation rate is very close to the mutation rate in isolates from a human transmission chain^26^. It has been reported that *Mtb* lineage 2 has higher mutation and drug resistance rates than *Mtb* lineage 4 in vitro^26^. Our results show that tested *Mtb* Beijing strains have high genomic mutation rates in vivo. The high genomic mutation rate is thus considered to be a characteristic of the *Mtb* Beijing strains.

Despite the valuable information obtained, we acknowledge the use of a small number of tested isolates as the first limitation to our study. However, it is comparable to the sample size used in the rhesus macaque study^16^ (based on four animals for active disease, three for latent and two for reactivation) and in the Rangipo strain outbreak in New Zealand (based on four pair-wise comparison)^17^. Nonetheless, our conclusion will need to be validated in larger studies, and analysis of different patient groups, including other Beijing strains from epidemic countries. Second, our study design slightly differed from that of the New Zealand cases^17^ where patients who developed TB 2 years after the first patient had TB, were categorized as the early-onset group. This might be the reason why higher rate of oxidative damage-related mutations during TB latency was not observed in the New Zealand cases^17^.

In summary, we reported higher mutation rate of *Mtb* Beijing strains during human infection. This higher mutation rate accounts for the higher adaptation ability and drug resistance rates of *Mtb* Beijing lineage. Currently, *Mtb* Beijing strains are the cause of multidrug-resistant tuberculosis worldwide, including Japan and are expanding. We suggest these biological factors should be considered in the efforts to limit the emergence of new resistance to both existing antibiotics and treatment regimens.

## Methods

### DNA extraction, library preparation and whole genome sequencing

Experiments were performed in class II safety cabinets in biosafety level 3 laboratories in Niigata University, Kobe Institute of Health, and Osaka Prefectural Institute of Public Health, to prevent accidents and contamination according to the institute’s guidelines. Genomic DNA of each Isolate A-F was prepared from colonies grown on Ogawa medium according to a previous report^47^. Other two *Mtb* Beijing isolates were inoculated on Ogawa egg medium and incubated at 35 °C until growth was observed. Genomic DNA of these two isolates were purified as a previous report^48^.

The library preparation and whole genome sequencing were performed at Dragon Genomics Center (Takara Bio, Shiga, Japan) or Beckman Coulter Genomics (MA, USA) where 75 bp paired-end reads were obtained by Illumina Genome AnalyzerIIx (Illumina, CA, USA).

### Mutation detection

The paired-end genome sequencing for the above eight strains was applied, and produced 16 FASTQ files. In order to retrieve high quality reads, usearch was performed with the parameter maxee 0.5 and 15–25% of reads were filtered. Each FASTQ file was mapped against *Mtb* H37Rv genome sequence, which was used as the reference genome, using BWA^49^. The resulting SAM files were transferred into the BAM format using samtools. Subsequently, SNPs and Indels were called with GATK^50,51^, generating the VCF files (Filter 1).

### Consensus sequence construction and mutation re-call

The low quality Indel and SNPs were filtered with the VCF parameters, quality by depth (QD) > 2, fisher strand (FS) > 60, root mean square of mapping quality (MQ) > 40, mapping quality rank sum test (MQ Rank Sum) equals to 0, allele count in genotypes (AC) equals to 2 and read position rank sum test (Read Pos Rank Sum) > − 8. In order to reconstruct the genome sequence for the first patient, Isolate A, the SNPs of the Isolate A were reflected to the *Mtb* H37Rv genome to generate the consensus genome sequence for the Isolate A using bcftools^52^. The indels were not applied in this process to avoid the complexity of the following process. The genome sequence for the five strains were re-mapped to the reconstructed reference genome sequence of Isolate A with the same method described above (Filter 2). Finally, indels re-called in Isolate A (similar to the Indels originally called using H37Rv as the reference) were removed in each strain (Filter 3). In order to further refine the re-called Indel and SNPs, the low quality Indel and SNPs were filtered with the same VCF parameters described above (Filter 4). Mutations found in the PE-PGRS family region were removed because they were found in the repeat sequence (Filter 5).

### Detection of large-deletion

Genomic regions with more than 1 kb that any reads were not mapped were found using our ad-hoc Ruby script. All the detected large-deletions were manually checked by the IGV genome viewer^53^ and the all detected deletions were confirmed to be deletions.

### Estimation of mutation rate

The incubation period for each strain was calculated based on the latency period from the onset of the first patient. As in Ford et al^16^, the mutation rate (*μ*) was calculated by the following equation.

$$\mu = \frac{m}{{N*\left( \frac{t}{g} \right)}}$$

*m* is defined by the number of mutations observed, *N* is the genome size based on the mapping coverage without PE-PGRS family region, *t* is the incubation period of each strain and *g* is the generation time. This generation time was set to the same 20 h as used in ref^16^. Mean reference genome coverage was 90.7% (range 89.1–91.4%).

### Phylogenetic analysis

The phylogenetic analysis was performed using CLC Genomics Workbench ver 20.0.2 (QIAGEN Aarthus A/S). All figures were derived from the resulting table generated by the software. After uploading the data files to the CLC server the sequence reads were trimmed to remove short and low quality reads (length > 20 bases, quality > 0.05) and then they were mapped to the *Mtb* H37Rv genome (NCBI accession #N000962)^54^. The evolutionary history was inferred using Maximum Likelihood algorism without bootstrap confidence values (1000 replications). The evolutionary distances were computed using the Jukes Cantor model and are represented as the number of base distances per site.

### Statistical analysis

We compared the number of mutations by Mann–Whitney U-test to analyze the bias of mutation positions. We compared the mutation rates of two groups by Student’s t-test using the R project for statistical computing. Differences were considered significant when the *p* value was ≤ 0.05.

### Accession codes

Sequencing reads have been submitted to the DNA Data Bank of Japan (DDBJ) under the study accession DRA010887. Accession numbers of 8 *Mtb* Beijing strains are listed in Supplementary Table S4.

## Supplementary information

Supplementary Information
