# Supplementary material for: Higher genome mutation rates of Beijing lineage of Mycobacterium tuberculosis during human infection
Source: Sci Rep. 2020 Oct 22;10:17997. doi: 10.1038/s41598-020-75028-2 (PMC7582865; doi:10.1038/s41598-020-75028-2)
Supplement: Supplementary file 1 — Supplementary Information [file 41598_2020_75028_MOESM1_ESM.docx]

**Supplementary Data**

**Higher genome mutation rates of Beijing lineage of *Mycobacterium tuberculosis* during human infection**

Mariko Hakamata, Hayato Takihara, Tomotada Iwamoto, Aki Tamaru, Atsushi Hashimoto, Takahiro Tanaka, Shaban A. Kaboso, Gebremichal Gebretsadik, Aleksandr Ilinov, Akira Yokoyama, Yuriko Ozeki, Akihito Nishiyama, Yoshitaka Tateishi, Hiroshi Moro, Toshiaki Kikuchi, Shujiro Okuda and Sohkichi Matsumoto

| Gene | Position | Variant | Isolate | | | | | | | | | | Annotation |  |
| --- | --- | --- | --- | --- | --- | --- | --- | --- | --- | --- | --- | --- | --- | --- |
|  |  |  | **A** | **B** | **C** | | **D** | | **E** | | **F** | |  | |
| Rv0014c | 16554 | synonymous | A | C | C | |  | |  | |  | | pknB; Transmembrane serine/threonine-protein kinase B PknB (protein kinase B) (STPK B) | |
| Rv0020c | 29629 | upstream | A | C | C | |  | |  | |  | | fhaA; Conserved protein with FHA domain, FhaA | |
| Rv0084 | 92343 | synonymous | T | G | G | | G | |  | |  | | hycD; Possible formate hydrogenlyase HycD (FHL) | |
| Rv0150c | 177503 | upstream | G | T | T | | T | | T | | T | | hypothetical protein | |
| Rv0199 | 236924 | synonymous | A | C | C | |  | |  | |  | | pknB; Transmembrane serine/threonine-protein kinase B PknB (protein kinase B) (STPK B) | |
| Rv0275c | 336422 | upstream | T | + | + | | + | |  | |  | | Probable conserved membrane protein | |
| Rv0282 | 342467 | synonymous | G | T | T | |  | | T | |  | | Possible transcriptional regulatory protein (possibly TetR-family) | |
| Rv0284 | 348006 | synonymous | G | T | T | | T | | T | | T | | eccA3; ESX conserved component EccA3 ESX-3 type VII secretion system protein | |
| Rv0315 | 383831 | synonymous | G | T | T | |  | |  | |  | | eccC3; ESX conserved component EccC3 ESX-3 type VII secretion system protein Possible membrane protein | |
| Rv0328 | 394372 | synonymous | T | G | G | |  | |  | |  | | Possible beta-1,3-glucanase precursor | |
| Rv0329c | 394987 | synonymous | A | C | C | |  | | C | |  | | Possible transcriptional regulatory protein (possibly TetR/AcrR-family) | |
| Rv0470c | 565479 | upstream | G | T | T | |  | | T | | T | | hypothetical protein | |
| Rv0541c | 633682 | synonymous | C | A | A | | A | | A | | A | | pcaA; Mycolic acid synthase PcaA (cyclopropane synthase) | |
| Rv0541c | 634022 | synonymous | C | A | A | | A | | A | | A | | Probable conserved integral membrane protein | |
| Rv0570 | 662268 | synonymous | A | C | C | |  | |  | |  | | Probable conserved integral membrane protein | |
| Rv0590A | 689004 | synonymous | T | G | G | |  | |  | | G | | nrdZ; Probable ribonucleoside-diphosphate reductase (large subunit) NrdZ (ribonucleotide reductase) | |
| Rv0613c | 708256 | synonymous | A | C | C | | C | | C | | C | | Mce-family related protein | |
| Rv0633c | 729338 | synonymous | T | G | G | |  | |  | |  | | hypothetical protein | |
| Rv0635 | 732359 | synonymous | T | G | G | | G | |  | | G | | Possible exported protein | |
| Rv0646c | 740707 | synonymous | A | C | C | |  | |  | |  | | hadA; (3R)-hydroxyacyl-ACP dehydratase subunit HadA | |
| Rv0667 | 762489 | synonymous | T | G | G | |  | |  | |  | | lipG; Probable lipase/esterase LipG | |
| Rv0779c | 872848 | synonymous | G | T | T | | T | | T | | T | | rpoB; DNA-directed RNA polymerase (beta chain) RpoB (transcriptase beta chain) (RNA polymerase beta subunit) | |
| Rv0790c | 884303 | synonymous | T | G | G | | G | | G | | G | | Possible conserved transmembrane protein | |
| Rv0791c | 885777 | synonymous | A | C | C | |  | |  | |  | | Hypothetical protein | |
| Rv0842 | 894548 | missense | T | G | G | |  | |  | |  | | hypothetical protein | |
| Rv0844c | 938940 | synonymous | C | A | A | | A | | A | | A | | Probable conserved integral membrane protein | |
| Rv0923c | 940880 | synonymous | T | G | G | |  | |  | |  | | narL; Possible nitrate/nitrite response transcriptional regulatory protein NarL | |
| Rv0932c | 1033803 | upstream | T | G | G | | G | |  | |  | | hypothetical protein | |
| Rv0941c | 1040594 | synonymous | A | C | C | |  | |  | |  | | pstS2; Periplasmic phosphate-binding lipoprotein PstS2 (PBP-2) (PstS2) | |
| Rv0989c | 1057311 | upstream | A | C | C | |  | |  | |  | | hypothetical protein | |
| Rv0992c | 1108561 | upstream | T | G | G | |  | |  | |  | | grcC2; Probable polyprenyl-diphosphate synthase GrcC2 (polyprenyl pyrophosphate synthetase) | |
| Rv1018c | 1108776 | synonymous | T | G | G | |  | |  | |  | | hypothetical protein | |
| Rv1022 | 1137805 | synonymous | T | G | G | | G | |  | |  | | glmU; Probable UDP-N-acetylglucosamine pyrophosphorylase GlmU | |
| Rv1030 | 1144251 | synonymous | G | T | T | | T | |  | | T | | lpqU; Probable conserved lipoprotein LpqU | |
| Rv1139c | 1154622 | synonymous | A | C | C | |  | |  | | C | | kdpB; Probable potassium-transporting P-type ATPase B chain KdpB (potassium-translocating ATPase B chain) (ATP phosphohydrolase [potassium-transporting] B chain) (potassium binding and translocating subunit B) | |
| Rv1155 | 1266961 | synonymous | A | C | C | |  | |  | |  | | hypothetical protein | |
| Rv1260 | 1281788 | synonymous | G | T | T | |  | |  | |  | | Possible pyridoxamine 5'-phosphate oxidase (PNP/PMP oxidase) (pyridoxinephosphate oxidase) (PNPOX) (pyridoxine 5'-phosphate oxidase) | |
| Rv1265 | 1296012 | synonymous | G | T | T | | T | | T | | T | | Probable oxidoreductase | |
| Rv1280c | 1408994 | synonymous | T | G | G | |  | |  | |  | | hypothetical protein | |
| Rv1309 | 1413774 | synonymous | A | C | C | | C | |  | |  | | oppA; Probable periplasmic oligopeptide-binding lipoprotein OppA | |
| Rv1356c | 1432814 | synonymous | T | G | G | |  | |  | |  | | atpG; Probable ATP synthase gamma chain AtpG | |
| Rv1394c | 1465795 | synonymous | G | T | T | | T | | T | | T | | Hypothetical protein | |
| Rv1529 | 1524316 | synonymous | A | C | C | |  | |  | |  | | cyp132; Probable cytochrome P450 132 Cyp132 | |
| Rv1547 | 1569774 | synonymous | A | C | C | |  | |  | |  | | fadD24; Probable fatty-acid-AMP ligase FadD24 (fatty-acid-AMP synthetase) (fatty-acid-AMP synthase) | |
| Rv1563c | 1729989 | synonymous | A | C | C | |  | |  | |  | | dnaE1; Probable DNA polymerase III (alpha chain) DnaE1 (DNA nucleotidyltransferase) | |
| Rv1631 | 1749748 | synonymous | A | C | C | |  | |  | |  | | treY; Maltooligosyltrehalose synthase TreY | |
| Rv1661 | 1768619 | synonymous | G | T | T | | T | | T | | T | | coaE; Probable dephospho-CoA kinase CoaE (dephosphocoenzyme a kinase) | |
| Rv1675c | 1835207 | synonymous | T | G | G | | G | | G | |  | | pks7; Probable polyketide synthase Pks7 | |
| Rv1722 | 1877235 | synonymous | A | C | C | |  | |  | |  | | cmr; Probable transcriptional regulatory protein Cmr | |
| Rv1727 | 1900571 | synonymous | T | G | G | |  | | G | |  | | Possible carboxylase | |
| Rv1751 | 1946746 | upstream | T | G | G | |  | |  | |  | | hypothetical protein | |
| Rv1823 | 1953660 | synonymous | T | G | G | |  | |  | |  | | Probable oxidoreductase | |
| Rv1835c | 1980680 | synonymous | T | G | G | |  | |  | |  | | hypothetical protein | |
| Rv1887 | 2070452 | synonymous | T | G | G | |  | | G | | G | | hypothetical protein | |
| Rv1947 | 2080953 | synonymous | T | G | G | |  | |  | |  | | Hypothetical protein | |
| Rv1963c | 2137068 | synonymous | A | C | C | | C | | C | |  | | Hypothetical protein | |
| Rv1966 | 2198177 | synonymous | T | G | G | |  | |  | |  | | mce3R; Probable transcriptional repressor (probably TetR-family) Mce3R | |
| Rv1982c | 2206274 | synonymous | A | C | C | | C | | C | |  | | mce3A; Mce-family protein Mce3A | |
| Rv2034 | 2210341 | synonymous | A | C | C | |  | |  | |  | | vapC36; Possible toxin VapC36 Contains PIN domain | |
| Rv2092c | 2225632 | synonymous | A | C | C | |  | |  | |  | | ArsR repressor protein | |
| Rv2093c | 2281460 | synonymous | T | + | + | |  | |  | |  | | helY; ATP-dependent DNA helicase HelY | |
| Rv2161c | 2351079 | synonymous | A | G | G | | G | | G | | G | | tatC; Sec-independent protein translocase transmembrane protein TatC | |
| Rv2180c | 2357268 | upstream | A | C | C | |  | |  | |  | | hypothetical protein | |
| Rv2250A | 2422360 | synonymous | G | GCGC | GCGC | |  | | GCGC | | GCGC | | Probable conserved integral membrane protein | |
| Rv2266 | 2442746 | synonymous | A | C | C | |  | |  | |  | | Possible flavoprotein | |
| Rv2351c | 2523205 | upstream | A | C | C | |  | |  | |  | | cyp124; Probable cytochrome P450 124 Cyp124 | |
| Rv2367c | 2540577 | synonymous | T | G | G | |  | |  | |  | | plcA; Membrane-associated phospholipase C 1 PlcA (MTP40 antigen) | |
| Rv2383c | 2631148 | synonymous | T | G | G | |  | |  | |  | | hypothetical protein | |
| Rv2519 | 2648421 | synonymous | T | G | G | |  | |  | |  | | mbtB; Phenyloxazoline synthase MbtB (phenyloxazoline synthetase) | |
| Rv2524c | 2675043 | synonymous | C | T | T | |  | | T | | T | | PE26; PE family protein PE26 | |
| Rv2531c | 2837226 | synonymous | A | C | C | |  | |  | |  | | fas; Probable fatty acid synthase Fas (fatty acid synthetase) | |
| Rv2540c | 2840836 | synonymous | T | C | C | | C | |  | |  | | adi; Probable amino acid decarboxylase | |
| Rv2614c | 2855706 | synonymous | A | C | C | |  | |  | |  | | aroF; Probable chorismate synthase AroF (5-enolpyruvylshikimate-3-phosphate phospholyase) | |
| Rv2739c | 2864102 | synonymous | A | G | G | | G | |  | |  | | thrS; Probable threonyl-tRNA synthetase ThrS (threonine-tRNA synthetase)(ThrRS) (threonine-tRNA ligase) | |
| Rv2781c | 2942394 | synonymous | C | A | A | | A | | A | | A | | Possible alanine rich transferase | |
| Rv2839c | 3052670 | synonymous | G | T | T | | T | |  | |  | | Possible alanine rich oxidoreductase | |
| Rv2898c | 3092943 | upstream | A | C | C | |  | |  | |  | | infB; Probable translation initiation factor if-2 InfB | |
| Rv2897c | 3147540 | synonymous | T | G | G | |  | |  | |  | | hypothetical protein | |
| Rv2968c | 3212135 | upstream | A | C | C | |  | | C | | C | | hypothetical protein | |
| Rv2978c | 3208268 | synonymous | A | C | C | |  | |  | | C | | Probable conserved integral membrane protein | |
| Rv2981c | 3323361 | synonymous | T | C |  | | C | | C | | C | | Probable transposase | |
| Rv3045 | 3327032 | synonymous | A | C | C | | C | | C | | C | | ddlA; Probable D-alanine--D-alanine ligase DdlA (D-alanylalanine synthetase) (D-ala-D-ala ligase) | |
| Rv3136A | 3334684 | synonymous | A | C | C | |  | |  | |  | | adhC; Probable NADP-dependent alcohol dehydrogenase AdhC | |
| Rv3195 | 3336825 | missense | A | C | C | |  | |  | |  | | hypothetical protein | |
| Rv3202c | 3406521 | synonymous | G | T | T | | T | | T | | T | | hypothetical protein | |
| Rv3239c | 3503229 | synonymous | G | T | T | | T | | T | | T | | Possible ATP-dependent DNA helicase | |
| Rv3270 | 3564633 | synonymous | G | T | T | |  | |  | |  | | Probable conserved transmembrane transport protein | |
| Rv3271c | 3579229 | synonymous | A | C | C | |  | |  | |  | | ctpC; Probable metal cation-transporting P-type ATPase C CtpC | |
| Rv3299c | 3617561 | synonymous | G | T | T | |  | |  | | T | | Probable conserved integral membrane protein | |
| Rv3332 | 3650836 | synonymous | G | T | T | |  | | T | | T | | atsB; Probable arylsulfatase AtsB (aryl-sulfate sulphohydrolase) (sulfatase) | |
| Rv3414c | 3653224 | synonymous | C | A | A | | A | |  | | A | | nagA; Probable N-acetylglucosamine-6-phosphate deacetylase NagA (GlcNAc 6-P deacetylase) | |
| Rv3455c | 3685441 | synonymous | G | A | A | |  | |  | |  | | sigD; Probable alternative RNA polymerase sigma-D factor SigD | |
| Rv3467 | 3719739 | synonymous | C | A | | A | | A | | A | | A | truA; Probable tRNA pseudouridine synthase a TruA (pseudouridylate synthase I) (pseudouridine synthase I) (uracil hydrolyase) | |
| Rv3468c | 3833050 | synonymous | C | + | | + | | + | |  | | + | hypothetical protein | |
| Rv3579c | 3846622 | upstream | T | A | | A | | A | | A | | A | rmlB3; Possible dTDP-glucose 4,6-dehydratase | |
| Rv3602c | 3846773 | upstream | G | C | | C | | C | | C | | C | Possible tRNA/rRNA methyltransferase | |
| Rv3645 | 3846775 | upstream | T | G | | G | |  | |  | |  | panC; Pantoate--beta-alanine ligase PanC (pantothenate synthetase) (pantoate activating enzyme) | |
| Rv3785 | 3846791 | upstream | C | + | |  | |  | |  | |  | Probable conserved transmembrane protein | |
| Rv3800c | 3851887 | missense | A | C | | C | | C | | C | |  | Hypothetical protein | |
| Rv3823c | 3851888 | synonymous | A | C | | C | |  | |  | | C | pks13; Polyketide synthase Pks13 | |
| Rv3833 | 3854553 | missense | C | G | | G | | G | | G | | G | mmpL8; Conserved integral membrane transport protein MmpL8 | |
| Rv3840 | 3880886 | upstream | A | C | | C | |  | |  | |  | Transcriptional regulatory protein (probably AraC-family) | |
| Rv3855 | 3884871 | missense | A | C | | C | |  | | C | | C | Possible transcriptional regulatory protein | |
| Rv3859c | 3890778 | upstream | T | G | | G | | G | |  | | G | ethR; Transcriptional regulatory repressor protein (TetR-family) EthR | |
| Rv3868 | 4021437 | synonymous | A | C | | C | |  | | C | | C | gltB; Probable ferredoxin-dependent glutamate synthase [NADPH] (large subunit) GltB (L-glutamate synthase) (L-glutamate synthetase) (NADH-glutamate synthase) (glutamate synthase (NADH))(NADPH-GOGAT) | |
| Rv3879c | 4044518 | synonymous | G | C | |  | |  | | C | | C | eccA1; ESX conserved component EccA1 ESX-1 type VII secretion system protein | |
| Rv3913 | 4083251 | synonymous | T | G | | G | |  | |  | |  | espK; ESX-1 secretion-associated protein EspK Alanine and proline rich protein | |
| Rv3424c | 4231859 | frameshift | G |  | | T | |  | |  | |  | trxB2; Probable thioredoxin reductase TrxB2 (TRXR) (TR) | |
| Rv3433c | 4231865 | synonymous | A |  | | C | |  | |  | |  | Hypothetical protein | |
| Rv3433c | 4260843 | synonymous | T |  | | C | |  | |  | |  | hypothetical protein | |
| Rv3785 | 4291046 | synonymous | C |  | | A | |  | |  | | A | hypothetical protein | |
| Rv2972c | 4307621 | synonymous | C |  | |  | | T | |  | |  | Hypothetical protein | |
| Rv3424c | 4313852 | synonymous | T |  | |  | | TGGA | |  | |  | Possible conserved membrane or exported protein | |
| Rv3424c | 4328193 | synonymous | A |  | |  | | + | |  | |  | Hypothetical protein | |
| Rv3424c | 4338565 | upstream | G |  | |  | | A | |  | |  | Hypothetical protein | |
| Rv0800 | 4344458 | synonymous | G |  | |  | |  | | T | | T | Hypothetical protein | |
| Rv1165 | 4359165 | synonymous | C |  | |  | |  | | A | | A | pepC; Probable aminopeptidase PepC | |
| Rv3435c | 4402448 | synonymous | C |  | |  | |  | | G | |  | typA; Possible GTP-binding translation elongation factor TypA (tyrosine phosphorylated protein A) (GTP-binding protein) | |

**Supplementary Table S1. Single nucleotide polymorphism identified in sequences isolates.**

| **Gene** | **Position** | **Variant** | **A** | **B** | **C** | **D** | **E** | **F** | **Annotation** |
| --- | --- | --- | --- | --- | --- | --- | --- | --- | --- |
| Rv2981c | 3336825 | missense | T | C (p.Thr365Ala) |  | C (p.Thr365Ala) | C (p.Thr365Ala) | C (p.Thr365Ala) | ddlA; Probable D-alanine--D-alanine ligase DdlA (D-alanylalanine synthetase) (D-ala-D-ala ligase) |
| Rv3467 | 3884871 | missense | C | A (p.Pro303His) | A (p.Pro303His) | A (p.Pro303His) | A (p.Pro303His) | A (p.Pro303His) | hypothetical protein |
| Rv3785 | 4231859 | frameshift | C | + (p.Gly186fs) |  | + (p.Gly186fs) | + (p.Gly186fs) |  | Hypothetical protein |
| Rv3433c | 3851887 | missense | A |  | C (p.Ser443Ala) |  |  |  | hypothetical protein |
| Rv0800 | 894548 | missense | G |  |  |  | T (p.Gly411Cys) | T (p.Gly411Cys) | pepC; Probable aminopeptidase PepC |
| Rv3435c | 3854553 | missense | C |  |  |  | G (p.Gly81Arg) |  | Probable conserved transmembrane protein |

**Supplementary Table S2. Single nucleotide polymorphism with amino acid substitution identified in sequences isolates.**

|  | Isolate |  |  |  |  |  |
| --- | --- | --- | --- | --- | --- | --- |
|  | B | C | D | E | F | H |
| Total | 107 | 108 | 47 | 48 | 46 | 46 |
| AT>GC | 4 | 4 | 5 | 3 | 3 | 7 |
| GC>AT | 2 | 2 | 2 | 1 | 1 | 3 |
| AT>CG | 69 | 70 | 13 | 13 | 13 | 15 |
| GC>TA | 23 | 25 | 16 | 18 | 21 | 20 |
| AT>TA | 1 | 1 | 1 | 1 | 1 | 0 |
| GC>CG | 3 | 2 | 2 | 4 | 3 | 1 |

**Supplementary Table S3. Number of different types of mutations among *Mtb* Beijing strains.**

| Isolate | Accession Number |
| --- | --- |
| A | DRX238935 |
| B | DRX238936 |
| C | DRX238937 |
| D | DRX238938 |
| E | DRX238939 |
| F | DRX238940 |
| G | DRX238941 |
| H | DRX238942 |

**Supplementary Table S4. The accession numbers of genome sequence data sequenced in this study.**

**
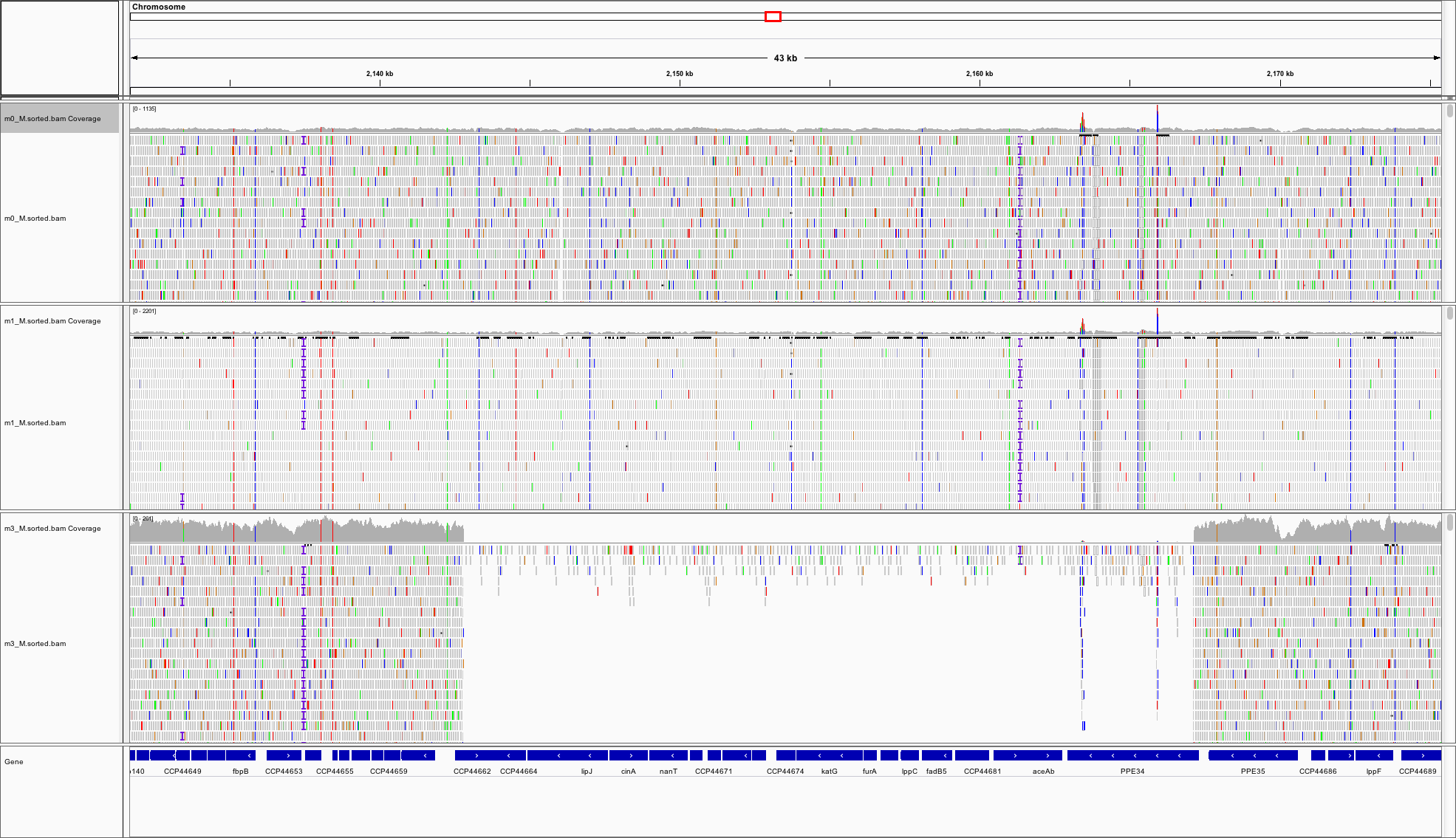
**

**Supplementary Figure S1. Example of detection of large deletion in Isolate D.** This figure shows the large deletion including *katG* in Isolate D.

**
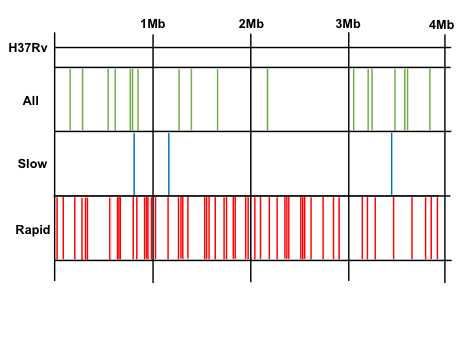
**

**Supplementary Figure S2. Location of the mutations in all strains, Slow and Rapid group.**

The lines indicate sites that have common SNPs among each all strains, SP and RP groups. The sites are only drawn approximately to scale.

**Supplementary Figure S3. Venn diagram showing the number of mutated genes shared by the New Zealand and Macaque study.**

The shared mutations with the New Zealand study by (17) Colangeli *et al*. and the Macaque study by (16) Ford *et al*. are described as Venn diagram.
